# Recent Investigations in the Physiological Functions of the Brain

**Published:** 1874-06

**Authors:** H. R. Bigelow

**Affiliations:** Hartford, Conn.


ART. II.—*Recent Investigations into the Physiological Functions of the Brain.* By H. R. BIGELOW, M. D., Hartford, Conn.

Some months ago I published in the *Canada Medical and Surgical Journal*, an article on the "Physiology and Psychology of the Brain," in which I endeavored, in a very general way, to reduce the modern facts, theoretrical and practical, relative to the Physiological and Metaphysical action of the Brain. We shall now be more concerned in the causation of paralyses, in the examination of the relations and functions of the cerebral centrifugal and centripetal nerve fibres, which transmit the force or convey the sensation, and in the consideration of the inter-relationship of the cerebral circulation, with the clinical evidences of pathological change. In the most essential particulars the views of yesterday are not those of to-day, yet the olden theories, not old in point of time, but merely as untenable premises are still tenaciously clung to, by many professional men. Professor Meynert* sees in the central nervous system a mechanism for receiving, storing up, and again transmitting, but not for originating excitations.

All excitations which affect the nervous system at all reach finally the cortex cerebri, upon which they may be said, in the geometrical sense, to be projected. The centripetal nerve fibres, through which this projection is accomplished, are included, together with the centrifugal fibres which place the Brain again in communication with the muscles, under the name of the "Projection System," in contradistinction to those systems of fibres which terminate with both their extremities in the cortex cerebri, and which serve to establish a functional unity between the different parts of that organ. These latter he calls the Association System. This article is full of brilliant hypotheses which it would be interesting to follow, but there is so much to reduce that the limits of an article will will hardly permit an extended review, and we pass to a consideration of the views which Dr. Brown-Sequard† considers well established, or most probable as regards the physiology and the physiological pathology of the Brain.

1.—The brain is a completely double organ, each of the hemispheres being a whole brain in itself, not only for mental actions as ably maintained by Sir Henry Holland, and by Dr. A. L. Wigan, but also for every other function (volition, intellectual perception of sensations, etc.) known to belong to the various parts of the central mass of nervous tissue named Encephalon.

2.—Originally no marked difference exists between the right and left hemispheres, each of them being in newly-born children almost as well able by natural development and future exercise to acquire all the functional powers of the brain; but as one of the two hemispheres being sufficient for certain functions, is exercised more than the other, it comes to be much more apt than the other to

* Stricker's Manual of Histology. J. J. Putnam's Summary, Archives Scientific Med., February, 1873.

† Archives of Scientific and Practical Medicine, Feb., 1873.

execute these functions. This view, ably maintained by Dr. W. Moxon, and accepted by Dr. P. Broca, so far as the faculty of speech is concerned, is just as well founded for the power of volition on the muscles, and for several other functions of the brain.

3.—The communications between muscles and brain as centres for volition take place in a normal state by two (if not more) distinct sets of conductors, one passing from a lateral half of the brain to the muscles of the corresponding side in the trunk and limbs, and remaining in all their length in the same side of the cerebro-spinal axis, the other set (or sets) decussating with the homologous set coming from the other side of the brain, going to the muscles of the trunk and limbs on the other side, (opposite.)

4.—The decussation of the conductors serving to voluntary movements takes place not entirely or even chiefly where most physiologists and physicians believe it to occur, i. e., in the lower and front part of the medulla oblongata; but all along the median plane of the cerebro-spinal centre, from the crura-cerebri to the lumbar extremity of the spinal cord, including, of course, the pons varolii and the medulla oblongata.

5.—The transmission of sensitive impressions, coming from the lateral halves of the body, takes place also by at least two sets of conductors, one going up to the brain in a direct way, i. e. remaining in the corresponding half of the spinal cord and of the brain, while the other passes into the other half of these nervous centres, decussating all along the spinal cord and medulla oblongata with the homologous fibres coming from the opposite side.

6.—The decussation of these last conductors takes place along the median plane of the spinal cord for the trunk and limbs, and of the encephalon for the nerves of general and special sensibility arising from that part of the nervous centres.

7.—Very few conductors, direct or decussating are sufficient for complete communication between the brain and spinal cord, for sensibility and voluntary movements. These communications take place not by mere prolongations of the nerve fibres of the motor and senitive roots of the spinal nerves, after they have reached the cells of the spinal cord, but by a very small number of conductors, going from groups of these last cells to groups of brain cells.

8.—The vaso-motor nerves arise from many parts of the nervous centres (including the gangloins of the sympathetic.) The medulla oblongata is the principal place of their decussation. The pons varolii is the chief centre from which they originate, either for the viscera or for the trunk and limbs.

9.—The respiratory movements have their centre in the spinal cord, the pons varolii and other parts of the encephalon, as well as in the medulla oblongata. Ganglions in the diaphragm participate in the production of these movements.

10.—Symptoms of diseases of the brain are of two distinct classes: those which arise from an influence exerted on distant parts by an irritation of the deceased part (indirect symptoms), and those produced in a direct way, either by the loss of function or the irritation of the part diseased. The first class will be observed in all cases of diseases of the brain which produce symptoms. This first class will co-exist with the second in most cases of diseases of the base of the brain; but it exists alone in cases of diseases located in the cerebral lobes, the cerebellum or its peduncles.

11.—The indirect symptoms are of two kinds, both arising, however, from an influence exerted on distant parts by an irritation starting from the organic lesion that we may see after death Of the first kind are those symptoms due to a paralyzing influence; and of the second kind are those in which, instead of cessation of activity, there is on the contrary the manifestation of a morbid activity.

12.—To the group of indirect (inhibitory or arrestatory) symptoms belong various kinds of cessation of activity, including the loss of voluntary movements, anæsthesia, amaurosis, aphasia, loss of consciousness, loss of the power of controlling the sphincters, etc.

13.—To the group of indirect production of a morbid activity belong delirium, convulsions and other active disorders of muscular movements.

14.—The mechanism of production of the indirect symptoms of an organic disease of the brain seems to be identical with that of the reflected symptoms due to the irritation of peripheric nerves in the bowels, the lungs, the skin, or in any other parts of the trunk and limbs.

15.—In the same way that an irritation of a peripheric nerve will sometimes produce no indirect symptom (i. e. a symptom due to an influence exerted on distant parts) or will in other instances produce any one of the indirect symptoms (those of cessation of activity, or those of production of a morbid activity,) in that same way will a lesion of the brain, extensive or not, produce no indirect symptom, or engender any one of the many indirect symptoms.

16.—The above being admitted, it is easy to understand all the differences that may be found as regards symptoms of organic diseases of the brain. It is easy to understand how a paralysis can occur on the side of the brain lesion or on the opposite side; how a paralysis can appear suddenly and be complete although the lesion is small, and located in a part which cannot by any one be considered as the seat of the will, or containing the conductors for the voluntary movements of the parts paralyzed; and how, also, a paralysis can disappear suddenly, although the brain lesion which gives rise to it still remains. It is easy to understand why there is no relation whatever between the extent, the kind, the seat and the rapidity of an organic disease of, or an injury to, the brain, and the symptoms that may appear. It is only in the acceptance of these views in their fullest sense, that we can at all hope to master clinical diagnosis. Hughlings Jackson* says: "Cases of paralysis and convulsion may be looked upon as the results of experiments made by disease on particular parts of the nervous system of man. The study of palsies and convulsions from this point of view is the study of the effects of "*destroying lesions*," and of the effects of "*discharging lesions*," and for an exact knowledge of the particular movements most represented in particular centres, we must observe and compare the effects of each kind of lesion. * * * * Limited *destroying lesions of some* parts of the cerebral hemisphere produce no obvious symptoms; whilst discharging lesions of those parts produce very striking symptoms. By this double method, we shall, I think, not only discover the particular parts of the nervous system where certain groups of movements are most represented (anatomical localization), but, what is of equal importance, we shall also learn the order of action (physiological localisation) in which

* Lancet, April, May, &c., 1873.

those movements are therein represented. I begin by speaking of destroying lesions, and take the simplest case—hemiplegia of the common form from lesion of the corpus striatum—a blood clot which has destroyed part of the corpus striatum has made an experiment, which reveals to us that movements of the face, tongue, arm and leg are represented in that centre. This is the localization of the movements anatomically stated. Physiologically we say that the patient whose face, tongue, arm and leg are paralyzed, has lost the most voluntary movements of one side of his body; it is equally important to keep in mind that he has not lost the more automatic movements. The study of hemiplegia shows that from disease of the corpus striatum those external parts suffer most which, physiologically speaking, are most under the command of the will, and which, physiologically speaking, have the greater number of different movements at the greater number of different intervals. That parts suffer more as they serve involuntary is, I believe, the law of *destroying* lesions of the cerebral nervous centres."

And again, "But there is proof that fibres pass from the *left* corpus striatum down into the *left* side of the cord, as well as into the *right* side; there are "direct" as well as "decussating" fibres. That there is a "decussating paralysis" from lesion of the left corpus striatum, no one doubts; but the existence of direct fibres, I think, supports the inference that there is also a transient "direct paralysis" from extensive lesion of that centre. After old lesions of the left corpus striatum there is Wallerian wasting of nerve fibres traceable from the seat of disease, not only down into the right side of the cord, but also into the left. This splitting of the bundle of wasted fibres on entering the cord is, I think, demonstrative evidence that *both* sides of the body are represented in the *left* corpus striatum. Does it not show that movements of the *left* face, arm and leg, are represented in the *left* corpus striatum by the *non-crossing* fibres, as well as that movements of the *right* face, arm and leg, are therein represented by the *crossing* fibres?

It may, however, be urged that these non-crossing fibres are solely for the bilaterally acting muscles ("muscles of the trunk.") But if we now consider the phenomena of a severe convulsion and find that from a discharging lesion of the left side of the brain the left face, arm and leg, are convulsed (after the right side) it is, I think, most reasonable to conclude that the non-crossing fibres are for the movements of the muscles of the left face, arm, and leg, although perhaps chiefly for those of the left side of the trunk." Hence we may conclude that both sides of the body are represented in each side of the brain, through this double arrangement of nerve fibre.

Having looked into this nervous co-relation, imperfectly I confess, but as thoroughly as a due regard for the economy of space and time will permit, I shall pass now to the review of some facts relating to the circulation of the brain, and its relation to apoplexy, syncope, &c.

The doctrine of the invariable quantity of blood within the cranium was first asserted by Dr. Alex. Monroe, of Edinburgh. He observes:* "As the substance of the brain, like that of the other solids of the body, is nearly incompressible, the quantity of blood within the head must be the same at all times, whether in health or disease, in life or after death, those cases only excepted in which water or other matter is effused or secreted from the blood vessels; for in these cases, a quantity of blood, equal in bulk to the effused matter, will be pressed out of the cranium."†

This view is essentially the same as that which Dr. Abercrombie, upon the authority of Dr. Viellie, long since propagated. These experiments of Dr. Viellie, and the inferences drawn for them, continued to obtain, until Dr. George Burrows, in his Lumleian Lectures, 1843–44, exposed the fallacy, and proved that the quantity of blood within the cranium, so far from being a constant or nearly constant quantity, is, on the contrary, as variable as in the other parts of the body, and the recent researches of Prof. His into the perivascular sheaths surrounding the cerebral vessels are corroborating proof. The numerous fissures and foramina for the transmission of nerves and vessels through the bones of the cranium, do away with the idea of the cranium being a perfect sphere like a glass globe.

We recognize certain cases of congestion, of anæmia, and we

* Observations, &c., on the Nervous System. Alexander Monroe, M. D., 1873.

† Dr. Abercrombie. Pathological and Practical Researches on Diseases of the Brain and Spinal Cord. Am Ed. p. 218.

know that in sleep the brain becomes anæmic, 'though Dr. Richardson has recently advanced the idea of natural sleep being caused by some molecular change in the cerebro-spinal system—this, however, requires further proof. The inhalation of chloride of amyl induces deep sleep and *anæmia* of the brain. Methylic ether causes deep sleep and *congestion* of the brain. The intra-cranial alterations give rise to certain intra-ocular appearances, which I shall fully describe under the head of the "opthalmoscope." The amount of vascular pressure within the brain is of great influence on the functions of the brain. Dr. Burrows* closes his article on this subject as follows:

"On this interesting and important principle of pressure, I have endeavored to point out that such a force is constantly in operation upon the cerebral substance; that this pressure is produced by vascular distension; that in health, any cause which is capable of increasing or diminishing this vascular distension has the effect of disturbing the functions of the brain; that these effects of vascular distension would be more serious and frequent if parts of the contents of the cranium were not readily removable upon increase of vascular pressure; that in pre-existing structural diseases of the encephalon, any increase of vascular distension causes much more serious disturbance of the cerebral functions, and the symptoms so produced are analgous to those of mechanical pressure on the brain. I have also attempted to support the opinion that variations of this vascular pressure are the causes of the intermitting character of the more urgent symptoms in cases of permanent disease within the cranium.

I have likewise endeavored to explain the phenomena of syncope, however produced, on the principle of diminished momentum of blood in the arteries of the head, and consequent diminished vascular pressure on the cerebral substance, rather than on the principle that the brain is not supplied with a sufficient quantity of blood.

And, lastly, I have accounted for many of the symptoms of disturbance of tne brain in general anæmea, upon the hypothesis of an insufficient vascular pressure on the substance of that organ."

* Burrows on Cerebral Circulation.

The same author says: "There are probably several causes capable of suspending the functions of the brain, and producing coma, and these causes are analogous to those which we experimentally find are adequate to destroy the functions of the cerebro-spinal nerves in any part of their course. These causes may be enumerated in the following order: First, pressure on the nervous fibres; secondly, division of the nervous substance; thirdly, disorganization of the nervous matter; fourthly, interrupted supply, or deficient momentum of blood in the nervous substance; and fifthly, the action of narcotics.

It appears to me that the true explanation of the cause of the coma in these cases of so-called simple apoplexy, is to be found in the previous existence of a state of congestion of the vessels within the cranium, brought on either by determination of blood to the head or detention of blood in that part. Then, as Dr. Watson has well expressed it, a tightening of the full vessels occasions extraordinary pressure on the nervous pulp; and hence the coma. But if this be the correct explanation of the production of the coma in the simple apoplexy of Abercrombie, why does the coma persist, and death so speedily ensue, although the vascular distension, the supposed cause of pressure, is removed by abstraction of blood, or other remedies, and, as we ascertain after death, the brain has sustained no structural lesion?

This is a question worthy of consideration. The fatal event is probably to be ascribed to another cause. If, in these attacks, the pressure on the brain has been adequate to suspend consciousness for a time, and the respiration has become altogether involuntary, slow, and stertorous, the substance of the brain is gradually saturated with undecarbonized blood. The apoplectic person remains in a condition analogous to that of one whose rima glottidis is constricted, or who has been suffering from apnœa for some time. The apoplectic person then dies, not simply from pressure or lesion of the brain, but from the effects of imperfect respiration."

If, then, the opthalmoscope reveal to us anæmia of the disk, we may be sure that there is a similar condition, a causative one, existing in the brain, and we must treat accordingly. So, also, if there be congestion, we should give strychnia or such drugs as will diminish this under determination.*

So also in simple aortic insufficiency, with or without hypertrophy of the left ventricle, there is spontaneous pulsation of the arteries of the disk and retina.

"The connecting nervous threads which run from the peripheral expansions of gray matter, constituting the sensory endowments of the skin, to the primary nuclei in the spinal cord, must in some manner be connected with the cortical layer in the cerebral lobes, otherwise it would be impossible for us to become conscious of the sensations which it is their function to convey. It is probable that this connection is made by secondary fibres, running from the spinal nuclei to the sensory-motor ganglia at the base of the brain, and that from these latter other fibres are developed which place the investing gray matter of the cerebral hemisphere in immediate communication with the corpora striata and optic thalami. The fact that the primary fibre from the integument to the cord terminates in the gray matter of the latter, and that in the other steps of the series communication is established through the connection of these nerve centres, does not invalidate the statement that there is a direct connection between the periphery and the supreme centres, although it renders it doubtful if this communication is made by one uninterrupted nerve thread.

The statement in regard to the connection between the superficial gray matter of the cerebrum and the peripheral expansions of nervous tissue which endow the integument with common sensibility to pain, is equally true in relation to the cerebral lobes and the motor nerves of muscles. An uninterrupted communication between the supreme centres and the contractile organs is necessary for the conscious performance of voluntary muscular acts. Yet this communication may be, and doubtless is, affected by the intervention of a number of secondary centres and fibres." †

This will explain the principle upon which the æsthesiometer is made use of in the Physical Diagnosis of Brain Disease; its construction and service will be dwelt upon at length in another paper.

---

* Otto Becker: London Ophth. Hos. Rep, Feb., 1873.

† Vance—Physical Diagnosis of Brain Disease. New York "Medical World," July, 1871.

To sum up then, in brief, some of the facts which, as skillful diagnosticians, we must be familiar with at the bedside, it is to be borne in mind.

1st. That grave cerebral lesions may exist without characteristic semeiology.

2d. That slight lesions may occasion a train of symptoms indicative of serious organic disease.

3d. That a paralysis is caused by the irritation transmitted by the cerebral lesion, inhibiting certain functional actions, and not by the mere circumscribed pressure.

4th. That a lesion in the middle line—i. e. affecting both sides of the optic thalmi, striate bodies, &c., may produce paralysis of but one side of the body.

5th. That a paralysis may exist on the same side with the lesion

6th. That unilateral convulsions are much oftener associated with lesion of the right brain than with the left.

7th. That hysterical paralysis &c., depends oftenest upon disorganized action of the right brain.

8th. That aphasia, agraphia, a mechanical impairment of speech and mental alienation, are usually associated with disease of the left brain.

9th. That the right brain is more capable than the left of producing a paralysis on the same or on the opposite side of the body.

10th. Optic neuritis is a cause of amaurosis depends more often on a disease of the right brain than on disease of the left.

11th. The symptoms may indicate a double lesion when one is present and conversely.

